# Implementation of a Cohort Retrieval System for Clinical Data Repositories Using the Observational Medical Outcomes Partnership Common Data Model: Proof-of-Concept System Validation

**DOI:** 10.2196/17376

**Published:** 2020-10-06

**Authors:** Sijia Liu, Yanshan Wang, Andrew Wen, Liwei Wang, Na Hong, Feichen Shen, Steven Bedrick, William Hersh, Hongfang Liu

**Affiliations:** 1 Department of Health Sciences Research Mayo Clinic Rochester, MN United States; 2 Department of Computer Science and Electrical Engineering Oregon Health & Science University Portland, OR United States; 3 Department of Medical Informatics and Clinical Epidemiology Oregon Health & Science University Portland, OR United States

**Keywords:** cohort retrieval, information retrieval, common data model, electronic health records, natural language processing

## Abstract

**Background:**

Widespread adoption of electronic health records has enabled the secondary use of electronic health record data for clinical research and health care delivery. Natural language processing techniques have shown promise in their capability to extract the information embedded in unstructured clinical data, and information retrieval techniques provide flexible and scalable solutions that can augment natural language processing systems for retrieving and ranking relevant records.

**Objective:**

In this paper, we present the implementation of a cohort retrieval system that can execute textual cohort selection queries on both structured data and unstructured text—Cohort Retrieval Enhanced by Analysis of Text from Electronic Health Records (CREATE).

**Methods:**

CREATE is a proof-of-concept system that leverages a combination of structured queries and information retrieval techniques on natural language processing results to improve cohort retrieval performance using the Observational Medical Outcomes Partnership Common Data Model to enhance model portability. The natural language processing component was used to extract common data model concepts from textual queries. We designed a hierarchical index to support the common data model concept search utilizing information retrieval techniques and frameworks.

**Results:**

Our case study on 5 cohort identification queries, evaluated using the precision at 5 information retrieval metric at both the patient-level and document-level, demonstrates that CREATE achieves a mean precision at 5 of 0.90, which outperforms systems using only structured data or only unstructured text with mean precision at 5 values of 0.54 and 0.74, respectively.

**Conclusions:**

The implementation and evaluation of Mayo Clinic Biobank data demonstrated that CREATE outperforms cohort retrieval systems that only use one of either structured data or unstructured text in complex textual cohort queries.

## Introduction

The widespread adoption of electronic health records has enabled the use of clinical data for clinical research and health care delivery [1]. Many institutions have established clinical data repositories in conjunction with cohort retrieval tools (eg, Informatics for Integrating Biology & the Bedside) to support the use of clinical data for research including retrospective studies as well as feasibility assessment or patient recruitment for clinical trials. However, electronic health record–based clinical research has been hampered by poor research reproducibility caused by the heterogeneity and complexity of both health care institutions and electronic health record systems.

For structured electronic health record data, to ensure a standardized and logically unified representation of electronic health record data across multiple institutions (and across multiple sites), many large-scale clinical research networks such as Accrual to Clinical Trials [2], Electronic Medical Records and Genomics [3], and National Patient-Centered Clinical Research [4] have adopted common data models aimed at producing comparable and reproducible results with the same research methods [5,6]. Our prior investigation [7] demonstrated the generalizability of one of the common data models, the Observational Medical Outcomes Partnership (OMOP) Common Data Model, in achieving structural and semantic consistency of electronic health record data in multi-institutional research with the Observational Health Data Sciences and Informatics (OHDSI) program [7].

In electronic health records, a significant portion of relevant patient information is embedded as unstructured text, and natural language processing techniques such as information extraction are critical when using these data for clinical research [8-11]. Many clinical natural language processing systems have been developed to extract information from text for various downstream applications [12,13] but have challenges in performance and portability [14-17]. Information retrieval, a technique used in search engines for storing, retrieving, and ranking documents from a large collection of text documents based on users’ queries, can provide an alternative approach to leverage clinical narratives for cohort retrieval as it is less semantic-dependent and can involve end users in the loop [18,19]. The combination of natural language processing and information retrieval is a promising solution for cohort retrieval from unstructured clinical text, and there are several review articles [5,20] about information retrieval or natural language processing techniques for case detection.

However, most of the current clinical data repository implementations do not support searches on both structured and unstructured text, seamlessly. An efficient and comprehensive patient-level search engine on both structured and unstructured data from electronic health record is, therefore, still highly demanded by health care practitioners and researchers. In this paper, we describe a proof-of-concept implementation of a cohort retrieval system—Cohort Retrieval Enhanced by Analysis of Text from Electronic Health Records (CREATE)—in which the same query to search both structured (electronic health record represented using the OMOP Common Data Model) and unstructured text (leveraging a concept extraction system) are used. Cohort retrieval in CREATE is conducted in 2 phases: the first phase filters patients using structured data, and the second phase retrieves and ranks results at either a document or a patient level. The functionality of the system was tested using a previously assembled query collection [21] on a corpus composed of the electronic health record data from the Mayo Clinic Biobank cohort [22].

There are generally 2 approaches to search unstructured text for purposes such as patient care, clinical research, and traceability of medical care [23]. The first approach is based on a text search. For example, the Electronic Medical Record Search Engine (EMERSE) from the University of Michigan [24] is a full-text search engine with the goal of facilitating the retrieval of information for clinicians, administrators, and clinical or translational researchers based on clinical narratives. However, EMERSE does not support queries using structured electronic health record data such as demographic information, lab tests, and medications. Dr. Warehouse, proposed by Garcelon et al [25], is a free-text search engine using Oracle Text to index its documents. The system is based on relational databases and relies on ranking after retrieval, which may limit its capability to deploy state-of-the-art information retrieval methods such as best match 25 or Markov random fields. The other approach to searching unstructured text is to extract concepts using natural language processing systems. For example, SemEHR [26] is a semantic search engine based on a Fast Healthcare Interoperability Resources [27] representation of clinical semantic concepts extracted from a clinical natural language processing system named Bio-YODIE. The system showed a high performance in retrieving patients given queries of single concepts, such as Hepatitis C and HIV, in local electronic health record and lab test results when evaluated with the MIMIC-III (Medical Information Mart for Intensive Care) data set [26].

National NLP Clinical Challenges 2018 (Shared-Task Track 1) [28] also contributed to standardized evaluations of cohort retrieval systems from electronic health records. The evaluation data set includes clinical narrative texts of 288 patients for concept extraction, temporal reasoning, and inferencing. The official evaluation indicated that the top systems used rule-based and hybrid systems for the problems and led the directions of future system development for similar tasks. The 2018 corpus consists of semistructured and narrative text. The structured data are provided via sections of semistructured text rather than in structured formats. Therefore, cohort retrieval systems for the 2018 corpus require additional components to handle the semistructured metadata, which may not be applicable to systems for real-world electronic health record data.

Several studies [29-31] have addressed the challenge of how to represent textual cohort criteria or queries via syntactic parsing or sequence labeling. The main focuses of proposed methods were to provide the functions of automatic parsing and modeling of textual queries of end-to-end retrieval systems. To further extend querying to support the customization of parsed results by end users, our cohort retrieval system has the following design principles: (1) the adoption of common data models to facilitate cohort retrieval using both structured and unstructured data for multi-institutional research, (2) the flexibility and ability to apply state-of-the-art information retrieval methods in the retrieval system, (3) the incorporation of relevance judgment for downstream machine learning–based cohort selection methods, and (4) the generation of semantic annotations during the indexing phrase to provide a real-time semantic search experience.

## Methods

### Overview of System Architecture

**Figure 1:**
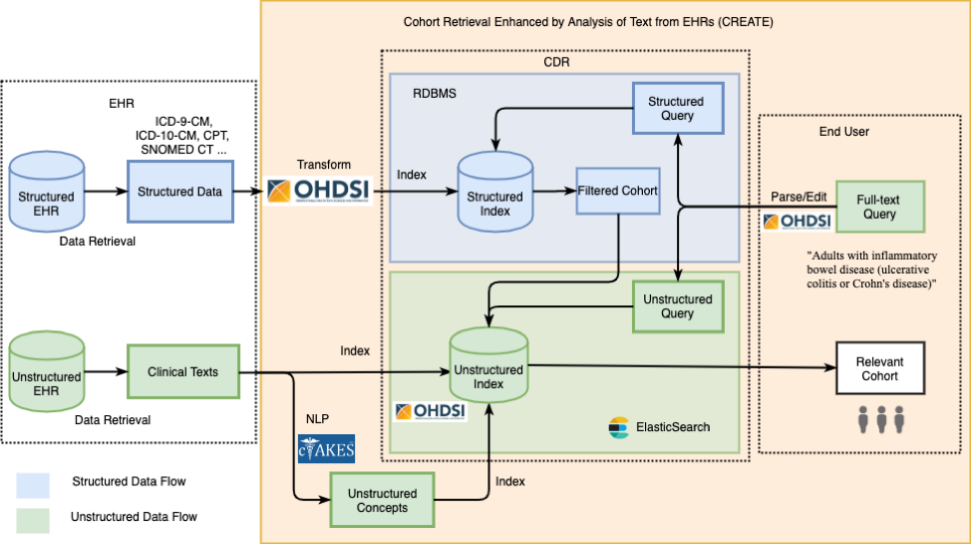
Overview of CREATE’s workflow. CDR: clinical data repository; CM: clinical modification; CPT: current procedures terminology; EHR: electronic health record; ICD: International Classification of Diseases; NLP: natural language processing; OHDSI: Observational Health Data Sciences and Informatics; RDBMS: relational database management system.

An overview of our cohort retrieval system for clinical data repositories is shown in Figure 1. Specifically, a textual query is expanded and divided, either automatically or manually, into structured and unstructured data fields according to specific clinical data repository implementations. The query fulfillment for structured data and unstructured text data are managed differently: structured electronic health record data can be retrieved from the corresponding clinical data repositories using Structured Query Language (SQL) on a relational database management system, and the unstructured electronic health record data can be preprocessed by natural language processing and retrieved by leveraging information retrieval techniques. Retrieved results can then be combined and aggregated for clinical research applications, such as clinical trial feasibility assessment or cohort identification. For cohort identification, the retrieved and screened cohort can be treated as a weakly labeled data set. Human relevance judgment is a potential subsequent step to manually validate the results through chart review.

### Adopting OMOP Common Data Model for Patient Retrieval

**Figure 2:**
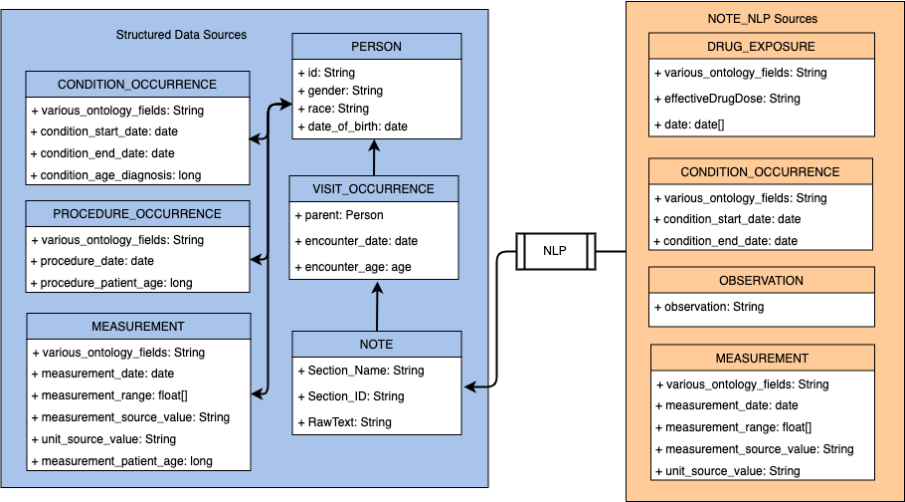
Hierarchical index structure using the OMOP Common Data Model. NLP: natural language processing.

To improve the interoperability and portability of our system (use with disparate data sources), we adopted the OMOP Common Data Model (version 5.3.1) [32] to index electronic health record data. The hierarchical index structure of clinical data repositories using OMOP Common Data Model for cohort retrieval is shown in Figure 2. The indexed tables include data from both unstructured and structured sources, consisting of extracted OMOP Common Data Model artifacts from unstructured clinical notes and encounter information, demographic information (represented as a common data model person), and diagnoses, procedures, and lab tests from structured data. The distinction between structured and unstructured data varies between different electronic health record systems. The specifics of implementation in adopters may, therefore, differ from those implemented in this study.

Structured data such as procedures, diagnoses, lab tests, and demographics are directly queried from relational databases and loaded into the index through an extract-transform-load process. We map structured data to Unified Medical Language System (UMLS) concept unique identifiers either through the usage of mapping definitions already in the UMLS Metathesaurus [33] (eg, ICD-9-CM or ICD-10-CM, Current Procedural Terminology 4, and SNOMED Clinical Terms) or through the use of natural language processing (eg, local lab test codes). The concepts are subsequently mapped to equivalent OHDSI- or OMOP-compliant vocabulary codes via Athena (version 1.10.0; OHDSI) standardized vocabularies [34].

The clinical texts from Mayo Clinic electronic health records consisted of existing sections that provide brief descriptions of a specific perspective from a patient encounter, such as social history, diagnosis, and chief complaints. We chose to use the document sections to index clinical text for cohort retrieval based on the observation that while retrieval at a sentence level is insufficient for relevance judging relevance in the topic collections that we investigated, document-level retrieval may provide mostly irrelevant information.

Various common data model concepts were extracted via the dictionary lookup component as entity mentions such as *Drug*, *Procedure*, and *SignSymptom* with their concept identifiers (eg, UMLS concept unique identifiers) by cTAKES (Apache Software Foundation) [12] from clinical documents and subsequently indexed into Elasticsearch (Elasticsearch BV). In addition, the entity mention attributes such as *negation*, *certainty*, and *family history* are stored in the field *term_modifiers*.

### Textual Query Formation

**Figure 3:**
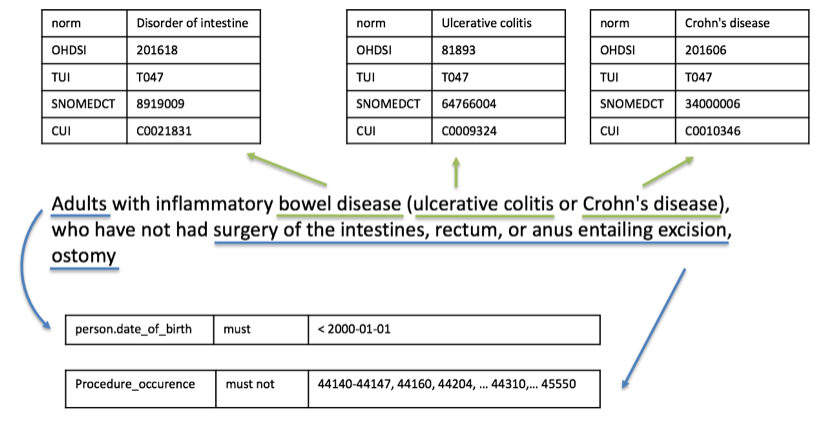
Textual query modeling example. CUI: concept unique identifier; OHDSI: Observational Health Data Sciences and Informatics; SNOMEDCT: Systematized Nomenclature of Medicine—Clinical Terms; TUI: (semantic) type unique identifier.

Natural language textual queries are fed into the same concept extraction pipeline used for indexing. Similarly, the normalized concepts and their associated attributes (eg, negation, certainty, experiencer, or status) are extracted from the textual query. Logical concepts such as *must* and *must not* are also used when generating queries from the text for further parsing and interpretation in the query backend. An example of the textual query modeling process is illustrated in Figure 3. In the query “Adults with inflammatory bowel disease (ulcerative colitis or Crohn's disease), who have not had surgery of the intestines, rectum, or anus entailing excision, ostomy,” the natural language processing component can detect and normalize the raw mentions of “bowel disease,” “ulcerative colitis,” and “Crohn’s disease” into various coding systems including OHDSI IDs, while the demographic information of “adults” and the list of surgeries can be manually added as structured data filters based on the date of birth and Current Procedural Terminology 4 codes.

### User Interface

We developed a web-based user interface for CREATE, the details of which are described in Multimedia Appendix 1. All the information extracted is shown to the users by subject for potential insertion, modification, and deletion before query execution. Since the natural language processing component will suggest parsing results and map them into common data model concepts, the users are expected to focus on configuring the logistics between the extracted concepts and removing generic concepts (eg, UMLS concepts of *Drug* or *Treatment*), which do not require searching concepts among standard vocabularies from various sources and are not time consuming.

### Retrieval Methods

CREATE uses Elasticsearch [35] as the search engine of the backend information retrieval component. Since Elasticsearch includes support for hierarchical queries of parent–child relations, the hierarchical index architecture shown in Figure 2 allows for significant flexibility in query strategies. Patients with a certain set of common data model concepts can be retrieved and filtered during the query execution by one of the structured fields (eg, encounter age), one of the unstructured fields (eg, whether the patient has sections containing common data model concepts from unstructured data), or both.

Given a document *d* and a textual query *q*, the set of common data model concepts extracted from *q* can be represented as *O* = {*o*_1_,...,*o*_n_} where *o* is a common data model concept. The similarity score between *d* and *o* can then be represented as *s*(*d*,*o*). The total score of each document for each query would then be defined as:

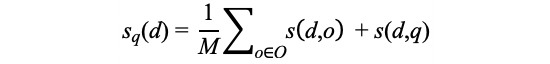



The first term on the right-hand side of the equation is the mean similarity of all common data model concepts in the query. The second term is the similarity between the document and the full-text query. In extreme use cases, the 2 terms can be weighted to place more emphasis on the contribution of either structured or unstructured data to the query. The patient-level similarity score is the mean of the top 100 document scores. The top rank threshold of 100 was selected based on our experiments on top 10, 20, 50, and 100 from test query results and may be subject to further tuning.

### Functionality Assessment of CREATE

There are 2 aspects of the system design that require feasibility assessment by real-world implementation for clinical data repository.

First, the data mapping needs to be created specifically for each clinical data repository architecture. A site-dependent correlation between clinical data repository representation, OMOP Common Data Model tables, and extracted natural language processing concepts has to be established before the data can be indexed into CREATE. Second, retrieved results need to be assessed to validate that the proposed query modeling and retrieval methods can generate meaningful retrieval results.

The performance was measured using the mean *precision at 5* of 5 queries. As an evaluation of CREATE functionality, we randomly sampled 5 queries from a previously curated query collection [21,36] to evaluate CREATE through manual chart review. The structured query used manually transformed ICD-9-CM or ICD-10-CM codes. There was no ranking of relevance for the retrieved patients from structured electronic health record data, thus we randomly selected 5 patients from the relevant patients to be used as the top 5 in calculating the precision at 5. The top 5 patients from unstructured text queries and CREATE results were retrieved based on best match 25 [30]. A medical expert performed complete chart review on the top 5 patients for each retrieval cohort. The patient relevancy was scored into the 3 categories, *definitely relevant*, *partially relevant*, and *not relevant*, by the medical expert. Definitely relevant, partially relevant, and not relevant were assigned scores of 1, 0.5, and 0, respectively, for precision at 5 calculations.

## Results

We implemented CREATE as a feasibility assessment tool for the Mayo Clinic Biobank Rochester cohort, which is a large-scale institutionally funded research resource initiated in 2009 with blood, electronic health record, and patient-provided data on 45,613 Mayo Clinic Rochester patients who had consented to participate. This resource has been used in a wide array of over 250 health-related research and clinical studies [22]. In our experiments, we limited inclusion to patients with at least one clinical note in their electronic health record and extracted the corresponding structured data.

After data extraction, we investigated and compared the electronic health record system implementation at the Mayo Clinic to OMOP Common Data Model tables. During the data exploration stage, we found that the data elements under corresponding tables were generally straightforward to map; therefore, we show the mapping at the granularity of the table level. Table 1 shows our mapping of several OMOP Common Data Model tables to Mayo Clinic electronic health record tables. The mapping used to transform named entity mention types of the cTAKES-type system to common data model tables is also listed in Table 1.

**Table 1:** Table-level mapping between OMOP Common Data Model and Mayo Clinic electronic health records.

OMOP^a^ Common Data Model and Mayo Clinic clinical data repository		Number of records	Vocabulary	Natural language processing cTAKES-type system
**Person**				
	Demographics	45,613	—^b^	—
**Condition**				
	Diagnosis	9,712,736	ICD-9-CM^c^ ICD-10-CM^d^	SignSymptom DiseaseDisorder
	Procedures	13,014,264	CPT^e^	Procedure
**Measurement**				
	Lab	15,719,203	Local code system	Lab
	Vital Signs	—	—	VitalSigns
**Drug_Exposure**				
	DrugExposure	—	UMLS^f^	Medication
**Note**				
	Clinical notes	68,198,499	—	—

^a^OMOP: Observational Medical Outcomes Partnership.

^b^There is no equivalent or no system is used for the equivalent concept.

^c^ICD-9-CM: International Classification of Diseases, Ninth revision, Clinical Modification.

^d^ICD-10-CM: International Classification of Diseases, Tenth revision, Clinical Modification.

^e^CPT: Current Procedural Terminology.

^f^UMLS: Unified Medical Language System.

Table 2 lists the detailed description of the 5 queries and the corresponding keywords used in the manual chart review process for judging patient relevance. The queries were different from the single condition criteria used to evaluate systems in some of the related work with regard to the level of detail, logic, and semantic complexity involved. The complete parsing results of the structured part of the queries and the CREATE query format specification can be found in Multimedia Appendix 2 and Multimedia Appendix 3, respectively.

Precision at 5 results are shown in Table 3. The overall comparison shows that CREATE, as a combination of systems using structured and unstructured electronic health record data, outperformed the systems based on using only one of structured or unstructured electronic health record data for full-text queries. For each query, CREATE performs at least as well as the systems using only structured or unstructured electronic health record data.

**Table 2:** The list of tested queries.

Query	Description	Keywords
1	Adults with inflammatory bowel disease (ulcerative colitis or Crohn's disease), who have not had surgery of the intestines, rectum, or anus entailing excision, ostomy	Ulcerative colitis, Crohn's disease, excision, ostomy, rectal prolapse, anal fistula, stricturoplasty resection
2	Adults 18-100 years old who have a diagnosis of hereditary hemorrhagic telangiectasia (HHT), which is also called Osler-Weber-Rendu syndrome.	Osler-Weber-Rendu syndrome, hereditary hemorrhagic telangiectasia
3	Children with localization-related (focal) epilepsy with simple or complex partial seizures diagnosed before 4 years old who have had an outpatient neurology visit.	Epilepsy, partial seizure, neurology
4	Adults 18-70 years old with rheumatoid arthritis currently treated with methotrexate who have never used a biologic disease-modifying antirheumatic drug (DMARD).	Rheumatoid arthritis biologic methotrexate abatacept, adalimumab, anakinra, certolizumab, etanercept, golimumab, infliximab, rituximab, tocilizumab, tofacitinib
5	Adults who have been treated with an angiotensin-converting-enzyme (ACE) inhibitor and developed an associated cough, consistent with ACE inhibitor–induced cough as an adverse effect of the medication.	Benazepril, Lotensin, Captopril, Enalapril, Vasotec, Fosinopril, Lisinopril, Prinivil, Zestril, Moexipril, Perindopril, Aceon, Quinapril, Accupril, Ramipril, Altace, Trandolapril, Mavik, cough, angiotensin-converting-enzyme (ACE) inhibitor

**Table 3:** Precision at 5 of sampled queries for electronic health record text.

Query	Structured	Unstructured	CREATE^a^ (unstructured and structured combined)
1	0.8	0.6	0.8
2	0.7	1.0	1.0
3	0.3	0.5	0.8
4	0.7	0.7	1.0
5	0.2	0.9	0.9
Mean	0.54	0.74	0.90

^a^CREATE: Cohort Retrieval Enhanced by Analysis of Text from Electronic Health Records.

## Discussion

### Principal Findings

CREATE is a proof-of-concept for leveraging the combination of structured queries and information retrieval techniques to improve cohort retrieval performance while adopting the OMOP Common Data Model to enhance model portability. The evaluation of the implementation using sample queries supports our hypothesis that using a combination of structured and unstructured electronic health record data outperforms a single-source system in determining the relevance, from an input query, of any given patient electronic health record data for a particular clinical application. CREATE was designed to improve the efficiency of judging patient relevance, by shifting from human-query judgment (pull) to system-feed judgment (push).

Intuitively, the nature of the queries and how the query-related data are presented in the clinical data repository significantly impact the performance of the data source queried (ie, structured, unstructured, and combined). For instance, one of the major concepts in query 5 is treatment with angiotensin-converting enzyme (ACE) inhibitors. It is effective for information retrieval methods on unstructured text to select patients with ACE inhibitor–related cough, as the keywords *ACE inhibitor* and *cough* usually co-occur in clinical text contexts as adverse drug events. In contrast, it is challenging for structured data queries in this experiment. In our clinical data repository, the medication information is present only as semistructured text generated by computerized provider order entry without normalization into structured data. Therefore, there is no reliable way to obtain the relevant cohort purely on structured data, which leads to very low relevancy of the retrieved cohort. Such a limitation is usually not critical when unstructured text are queried, because most of the clinical data are either presented or summarized in clinical notes.

However, when querying on a cohort with age or gender criteria, querying solely on unstructured data cannot work effectively. For example, even when the age is mentioned in query 3, all the retrieved patients are adult patients rather than the expected pediatric patients. This is caused by the lack of the extraction the dates and ages from narrative texts, which is not a trivial information extraction task. To build a reliable query system for unstructured texts without providing metadata, such as date of birth or age at encounter, usually requires corpus-dependent engineering efforts to extract the dates and ages from narrative text.

### Limitations

This study has multiple limitations that may offer directions for our future work. Our current functionality test is based on 5-query precision at 5 by one annotator, which is not sufficient to cover all cohort retrieval cases and longitudinal patient condition scenarios. Though we acknowledge that a larger number of queries on a fully-annotated patient cohort from annotators and adjudicators would be very helpful in evaluating the performance of the system, it is time consuming to judge complete patient history, especially for negated conditions and treatments (eg, to check if the patient does not have a certain disorder or procedure). With the system in production, the feedback of each study leveraging the system can be then retained and analyzed for more comprehensive statistics of the performance of the system.

When processing concepts without a global coding system, concept mapping, such as that used in our solution, relies on the output of natural language processing algorithms. Although it is a fast and straightforward solution, current natural language processing tools cannot achieve the same level of accuracy as human assigned codes. Complete mapping from a local vocabulary requires extensive human efforts with data quality assurance [37], thus it was not feasible within the scope of this study. A solution for this issue is to utilize value set repositories to manage the concepts. Though a one-to-one mapping may not be found in all semantic spaces, value set repositories can provide a systematic way to manage the concept sets in collections or aggregations [38].

There are also several potential approaches to further improve the information retrieval component in this system's framework. We only used the out-of-box query algorithms to measure the patient similarity and rank the relevancy in this study. More advanced information retrieval methods can be applied to the queries such as case-based reasoning [39-41], pseudo relevance feedback [42], and different ranking models [43,44]. Though the equal weights of common data model concepts and raw text provide information from both sides, the weights can be tuned to meet different retrieval perspectives and demands.

### Conclusion

We developed CREATE, an end-to-end patient-level information retrieval system, with the ability to query both structured and unstructured data by leveraging the OMOP Common Data Model. Implementation and functionality assessment on Mayo Clinic Biobank demonstrated that CREATE outperforms cohort retrieval systems that use only one of either structured or unstructured data in complex textual cohort queries. The source code of the CREATE can be found at Multimedia Appendix 4.

In the future, we will refine the evaluation process by adding more query topics and larger cohort of manual chart reviews. An active learning component will be added to the system to enable human-in-the-loop analysis on the system-screened cohort to further improve the efficiency of relevance judgment. In doing so, both machine learning–based or rule-based cohort identification algorithms could be deployed and evaluated in real time. This could potentially then be extended to an active-learning cohort-identification framework [45].

## Appendix

Multimedia Appendix 1 Web-based graphical user interface of CREATE.

Multimedia Appendix 2 Parsing results of CREATE from textual queries.

Multimedia Appendix 3 Format specification for CREATE queries.

Multimedia Appendix 4 Source code of CREATE.

## Abbreviations

CREATE: Cohort Retrieval Enhanced by Analysis of Text from Electronic Health Records
EMERSE: Electronic Medical Record Search Engine
ICD-9-CM: International Classification of Diseases, Ninth Revision, Clinical Modification
ICD-10-CM: International Classification of Diseases, Tenth Revision, Clinical Modification
OHDSI: Observational Health Data Sciences and Informatics
OMOP: Observational Medical Outcomes Partnership
SNOMED: Systematized Nomenclature of Medicine
UMLS: Unified Medical Language System
