# Supplementary material for: Implementation of a Cohort Retrieval System for Clinical Data Repositories Using the Observational Medical Outcomes Partnership Common Data Model: Proof-of-Concept System Validation
Source: JMIR Med Inform. 2020 Oct 6;8(10):e17376. doi: 10.2196/17376 (PMC7576539; doi:10.2196/17376)
Supplement: Multimedia Appendix 1 [file medinform_v8i10e17376_app1.docx]

# Appendix 1: Web-based GUI of CREATE

We designed a web-based graphical user interface (GUI) of the CDM query builder. The GUI consists of three components: a query text box, a concept criteria editor and a CDM viewer. The query text box is similar with the search boxes of any search engines, providing the user a real-time search experience on CDM. The users can compose SQL-like queries on structured data filter. Figure 1 shows the UI of query editor on the full-text query “*Adults with inflammatory bowel disease (ulcerative colitis or Crohn's disease), who have not had surgery of the intestines, rectum, or anus entailing excision, ostomy*”. The builder can be used to include the inclusion and exclusion criteria on structured fields listed in the CDM. In this case, the age information regarding “adult” is manually transformed into date of birth. The diagnosis “*ulcerative colitis*” is translated into a list of ICD9 codes (e.g. “*556.**”). Then the exclusion criteria are used as the negation to exclude all patients with CDM concept with CPT codes in procedures. The second phase is to review the automatic extracted CDM concepts in CDM editor shown in the right of Figure 1. CDM concepts can be added or removed. Once both the structured CDM criteria and unstructured CDM concepts are reviewed and approved by users, the queries can be submitted.


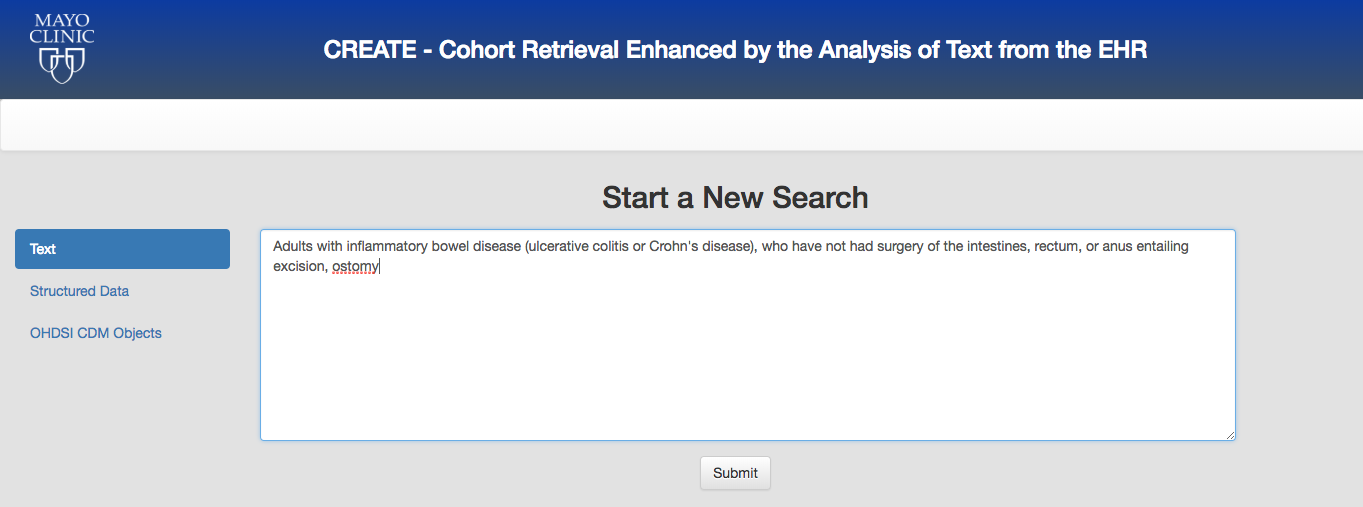


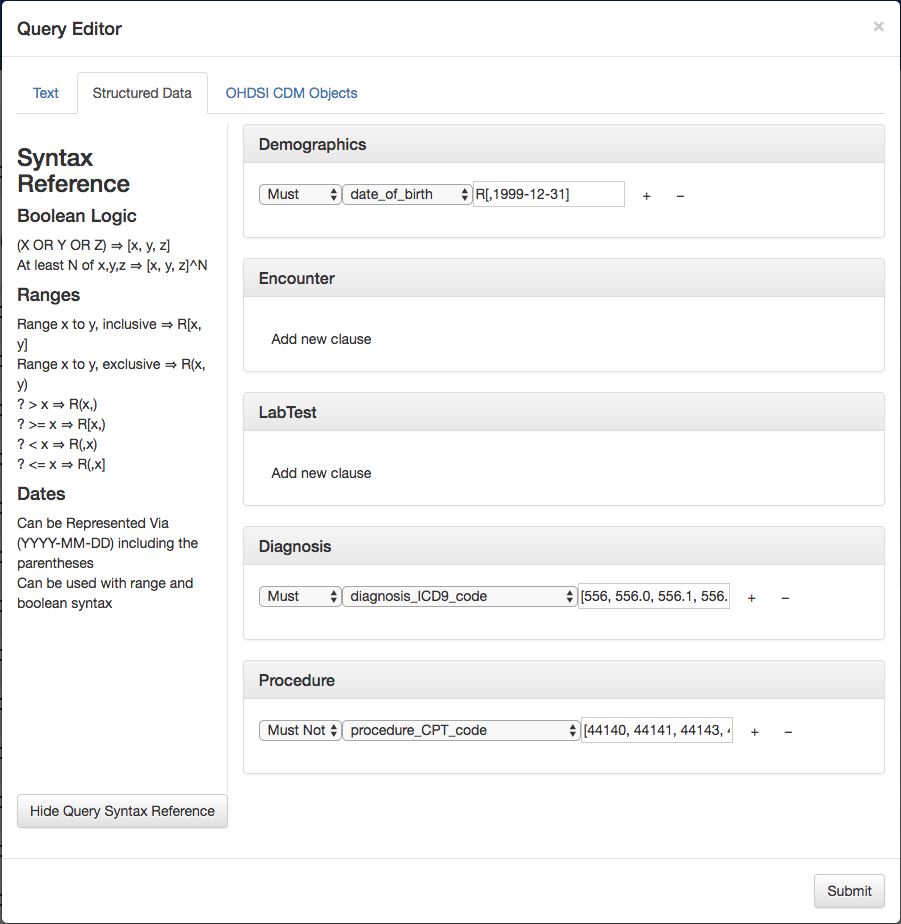

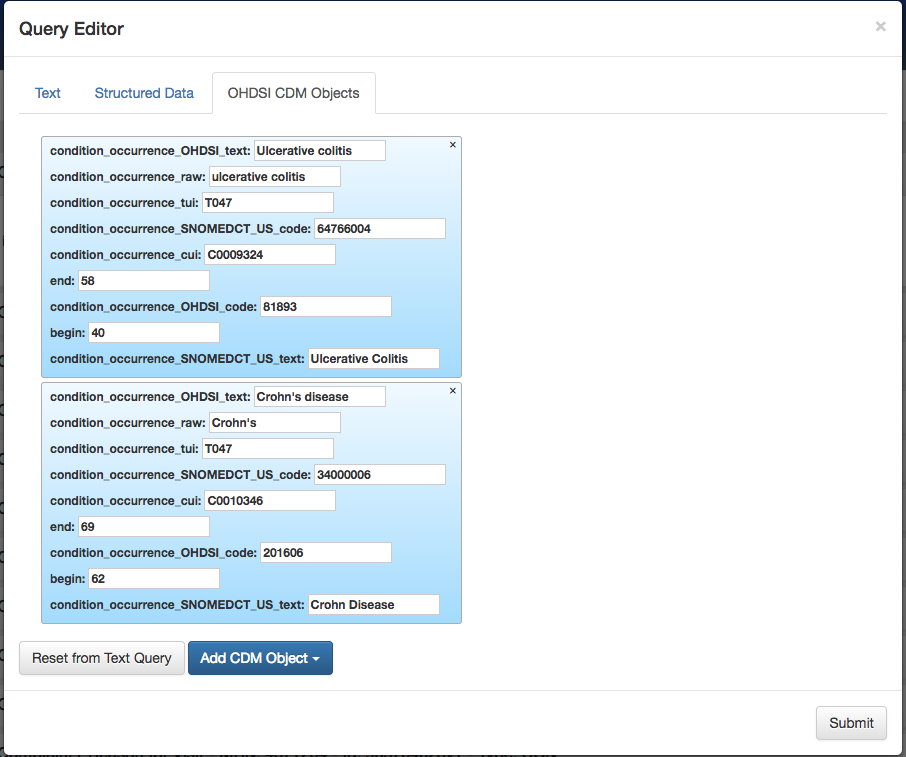


Figure 1 GUI of textual query editor (top), CDM Query Editor for structured query (bottom left) and OMOP CDM objects (bottom right) of query “*Adults with inflammatory bowel disease (ulcerative colitis or Crohn's disease), who have not had surgery of the intestines, rectum, or anus entailing excision, ostomy*”

Retrieval results are shown in the same page below the query composing modules as Figure 2. The interface includes two views: patient-level view and document-level view, which can be switched without rerunning the query. The two views serve different applications: patient-level view is intended for patient level retrieval or cohort identification tasks, and thus are more relevant to this study. Document-level views can be used for other general document level information retrieval tasks. Each retrieved patient or document has judgment buttons to conduct relevance judgment for quality measurement. The UI has the ability to save both the retrieval results and judgment results for later use.


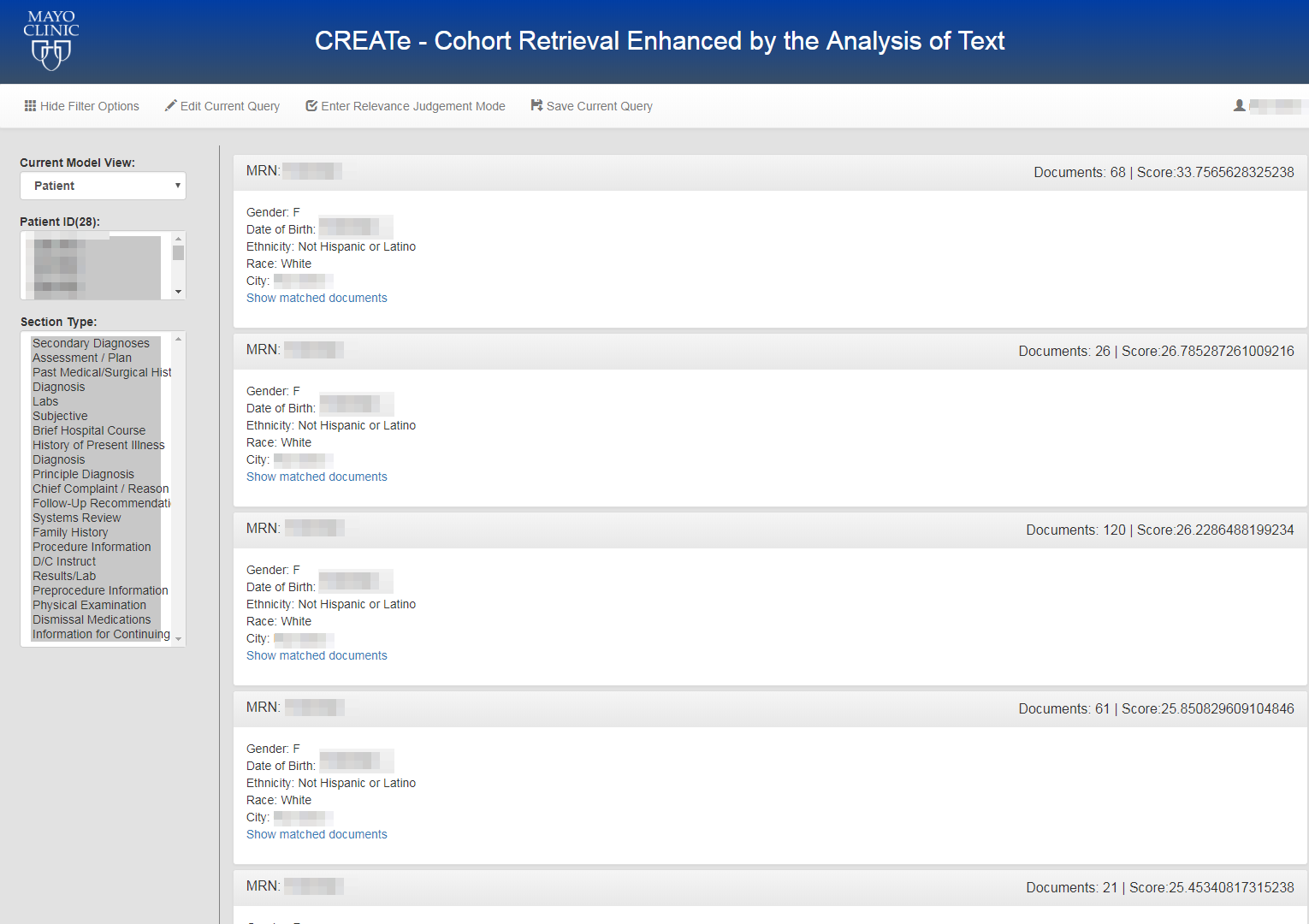

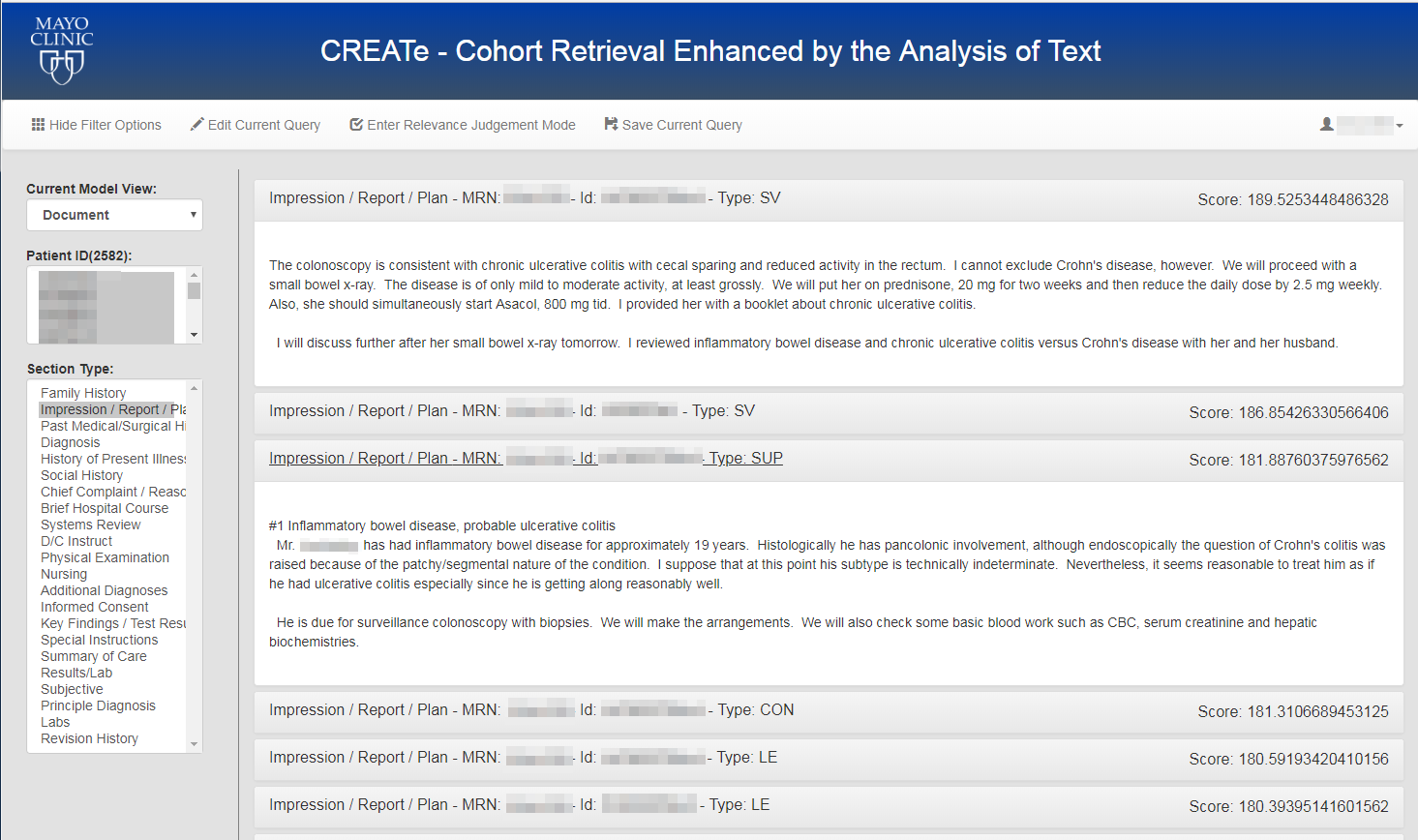


Figure 2 Retrieval results in patient view (top) and document view (bottom). MRN (Mayo Rochester Number) is equivalent to patient ID. ID in the document view refers to document ID.
