# Supplementary material for: Implementation of a Cohort Retrieval System for Clinical Data Repositories Using the Observational Medical Outcomes Partnership Common Data Model: Proof-of-Concept System Validation
Source: JMIR Med Inform. 2020 Oct 6;8(10):e17376. doi: 10.2196/17376 (PMC7576539; doi:10.2196/17376)
Supplement: Multimedia Appendix 2 [file medinform_v8i10e17376_app2.docx]

Appendix 2: Parsing results of CREATE from textual queries

The parsing results for structured queries in the topics following the specification in Appendix C.

## Topic 1

Adults with inflammatory bowel disease (ulcerative colitis or Crohn's disease), who have not had surgery of the intestines, rectum, or anus entailing excision, ostomy

| # -Demographics-  type: Person  +date_of_birth: R[ , 1999-12-31]  # -Diagnosis-  type: Diagnosis  # ulcerative colitis for first 11 elements or crohns for next 5 elements or other colitis for last element  + diagnosis_ICD9_code: [556, 556.0, 556.1, 556.2, 556.3, 556.4, 556.5, 556.6, 556.7, 556.8, 556.9, 555, 555.0, 555.1, 555.2, 555.9, 558.9]  # -Surgery-  type: Procedure  # colectomy  - procedure_CPT_code: [44140, 44141, 44143, 44144, 44145, 44146, 44147, 44160, 44204, 44205, 44206, 44207, 44208, 44150, 44151, 44155, 44156, 44157, 44158, 44210, 44212, 44211]  # enterectomy  - procedure_CPT_code: [44120, 44121, 44125, 44202, 44203]  # intestinal ostomy  - procedure_CPT_code: [44310, 44316, 44187, 44320, 44322, 44188]  # proctoplasty or proctoplexy  - procedure_CPT_code: [45500, 45505, 45540, 45541, 45550, 45400, 45402]  # stricturoplasty  - procedure_CPT_code: [44615, 45150, 46700]  # anal fistula  - procedure_CPT_code: [46270, 46275, 46280, 46285, 46288] |
| --- |

## Topic 2

Adults 18-100 years old who have a diagnosis of hereditary hemorrhagic telangiectasia (HHT), which is also called Osler-Weber-Rendu syndrome.

| type: Person  +BIRTH_DATE: R[1917-01-01, 1999-12-31]  # -Diagnosis-  diagnosis_ICD9_code: 448.0 |
| --- |

## Topic 3

Children with localization-related (focal) epilepsy with simple or complex partial seizures diagnosed before 4 years old who have had an outpatient neurology visit.

| # -Demographics-  type: Person  +date_of_birth: R[2000-01-01, ]  # -Diagnosis-  type: Diagnosis  diagnosis_ICD9_code: [345.40, 345.41, 345.50, 345.51, 780.39] |
| --- |

## Topic 4

Adults 18-70 years old with rheumatoid arthritis currently treated with methotrexate who have never used a biologic disease-modifying antirheumatic drug (DMARD).

| # -Demographics-  type: demographics  +BIRTH_DATE: R[1943-01-01, 1995-12-31]  # -Diagnosis-  type: Diagnosis  diagnosis_ICD9_code: 714.0  # -Drug_Exposure-  type: medications_ordered  # methotrexate  RX_DESCRIPTION: ["METHOTREXATE", "RHEUMATREX", "OTREXUP", "RASUVO", "TREXALL"]  RX_DISPLAY_NAME: ["METHOTREXATE", "RHEUMATREX", "OTREXUP", "RASUVO", "TREXALL"]  # tnf biologic  ~RX_DESCRIPTION: ["ADALIMUMAB", "HUMIRA", "CERTOLIZUMAB", "CIMZIA", "ETANERCEPT", "ENBREL", "GOLIMUMAB", "SIMPONI", "INFLIXIMAB", "REMICADE"]  ~RX_DISPLAY_NAME: ["ADALIMUMAB", "HUMIRA", "CERTOLIZUMAB", "CIMZIA", "ETANERCEPT", "ENBREL", "GOLIMUMAB", "SIMPONI", "INFLIXIMAB", "REMICADE"]  # non-tnf biologic  ~RX_DESCRIPTION: ["ABATACEPT", "ORENCIA", "RITUXIMAB", "RITUXAN", "ANAKINRA", "KINERET"]  ~RX_DISPLAY_NAME: ["ABATACEPT", "ORENCIA", "RITUXIMAB", "RITUXAN", "ANAKINRA", "KINERET"] |
| --- |

## Topic 5

Adults who have been treated with an angiotensin-converting-enzyme (ACE) inhibitor and developed an associated cough, consistent with ACE inhibitor-induced cough as an adverse effect of the medication.

| # -Demographics-  type: Person  + date_of_birth: R[, 1995-12-31]  # -Drug_Exposure-  type: Drug_Exposure  GENERIC_NAME_1: ["QUINAPRIL", "BENAZEPRIL", "LISINOPRIL", "CAPTOPRIL", "ENALAPRIL", "FOSINOPRIL", "RAMIPRIL", "PERINDOPRIL", "TRANDOLAPRIL", "MOEXIPRIL"]  GENERIC_NAME_2: ["QUINAPRIL", "BENAZEPRIL", "LISINOPRIL", "CAPTOPRIL", "ENALAPRIL", "FOSINOPRIL", "RAMIPRIL", "PERINDOPRIL", "TRANDOLAPRIL", "MOEXIPRIL"]  MED_NAME: ["QUINAPRIL", "ACCUPRIL", "BENAZEPRIL", "LOTENSIN", "LISINOPRIL", "PRINIVIL", "ZESTRIL", "CAPTOPRIL", "CAPOTEN", "ENALAPRIL", "VASOTEC", "FOSINOPRIL", "MONOPRIL", "RAMIPRIL", "ALTACE", "PERINDOPRIL", "ACEON", "TRANDOLAPRIL", "MAVIK", "MOEXIPRIL", "UNIVASC"] |
| --- |
