# Supplementary material for: Implementation of a Cohort Retrieval System for Clinical Data Repositories Using the Observational Medical Outcomes Partnership Common Data Model: Proof-of-Concept System Validation
Source: JMIR Med Inform. 2020 Oct 6;8(10):e17376. doi: 10.2196/17376 (PMC7576539; doi:10.2196/17376)
Supplement: Multimedia Appendix 3 [file medinform_v8i10e17376_app3.docx]

# Appendix 3: Format Specification for CREATE queries

The semi-structured query files use a specific format, appropriately referred to as the semi-structured query format. Using this format maintains consistency, and is essential to follow so that the file is parsed correctly.

### NAMING CONVENTIONS

#### Field

The field is designated as its specific name in Elasticsearch, which should match the Elasticsearch name and case (UPPERCASE). Indent by 4 spaces on a line under its corresponding document (to indicate the field is for that document)

#### Values

In general, follow the format in Elasticsearch. Use special characters preceding with \,

#### Dates

yyyy-mm-dd

Text

Use UPPERCASE for values (as convention). Put each value (phrase) in quotations if listing multiple values. Text values will match as the whole phrase.

### LOGIC CONVENTIONS

#### Fields

AND operator (filter)

+FIELD_NAME indicates required value

-FIELD_NAME indicates required negated value

OR operator (no filter)

FIELD_NAME indicates preferred value (no symbol before the field name)

~FIELD_NAME indicates preferred negated value

#### Values

OR operator

[x, y, z] indicates (x OR y OR z)

Can also specify that a certain number of values be present

[x, y, z]^2 indicates that at least 2 of (x OR y OR z) be present (always indicates 'at least', not 'only')

#### Ranges

Use "R" before the range to indicate it's a range and not a list of "OR" values

Use [] and () to indicate inclusivity or exclusivity of values

R[x, y] indicates range inclusive of x and y values

R(x, y) indicates range exclusive of x and y values

Can have one side inclusive and one exclusive

R[x, y) indicates inclusion of x up to but not including y

R(x, y] indicates exclusion of x but inclusion of values up to and including y

Greater than or lesser than

Specified as a range with one empty value

Use [] and () for inclusivity and exclusivity in the same manner as other ranges

R(x, ) indicates > x

R[x, ) indicates >= x

R(, x) indicates < x

R(, x] indicates <= x

#### Dates

Operations using dates usually require the date to be enclosed in parentheses

#### Range

Date ranges follow the same conventions as other ranges except that each date must be enclosed in parentheses. R[(yyyy-mm-dd), (YYYY-MM-DD)] indicates date yyyy-mm-dd (inclusive) up to and including date YYYY-MM-DD. R((yyyy-mm-dd), (YYYY-MM-DD)) indicates all dates between (but not including) dates yyyy-mm-dd and YYYY-MM-DD. Other combinations of [] and () as used in other ranges

#### OR operator

[(yyyy-mm-dd), (YYYY-MM-DD)] indicates (date yyyy-mm-dd OR date YYYY-MM-DD)

#### Multiple fields and values

Enclose in [] to list different fields and values in the same way as the OR operator for values.
