# Supplementary material for: Implementation of a Cohort Retrieval System for Clinical Data Repositories Using the Observational Medical Outcomes Partnership Common Data Model: Proof-of-Concept System Validation
Source: JMIR Med Inform. 2020 Oct 6;8(10):e17376. doi: 10.2196/17376 (PMC7576539; doi:10.2196/17376)
Supplement: Multimedia Appendix 4 [file medinform_v8i10e17376_app4.docx]

The source code is made available at <https://github.com/OHNLPIR/OMOP_CDM_IO>.
